# The Correlation Between Blood Group Type and Diabetes Mellitus Type II: A Case-Control Observational Study From Pakistan

**DOI:** 10.7759/cureus.19898

**Published:** 2021-11-25

**Authors:** Simrah Sharjeel, Muhammad Wasi, Aliya Jafri, Fatima A Raza, Zara Tariq, Khizer Shamim, Kiran Abbas, Moiz Ahmed

**Affiliations:** 1 Department of Medicine, Jinnah Sindh Medical University, Karachi, PAK; 2 Department of Biochemistry, Jinnah Sindh Medical University, Karachi, PAK; 3 Department of Medicine, Karachi Medical and Dental College, Karachi, PAK; 4 Department of Medicine, Ziauddin University, Karachi, PAK; 5 Department of Medicine, Jinnah Postgraduate Medical Centre, Karachi, PAK; 6 Department of Medicine and Surgery, Sindh Medical College, Karachi, PAK

**Keywords:** endocrinology, diabetes mellitus, abo blood group, blood, adult-onset type 2 diabetes mellitus

## Abstract

Introduction

Adult-onset type 2 diabetes mellitus (T2DM) is defined as a chronic hyperglycemic state, characterized by insulin resistance and declining islet B-cell function, eventually leading to islet B-cell function failure. The present study evaluated the association of T2DM with the type of blood group.

Methodology

A case-control study was conducted from April 2020 to September 2021 in Karachi, Pakistan. An electronic questionnaire was used to determine if there is an association between ABO blood groups and type 2 diabetes mellitus. Our study involved two groups with an equal number of participants. The patient group contained participants who had been diagnosed with type 2 diabetes mellitus, whereas the control group contained participants who had never been diagnosed with type 2 diabetes mellitus. Information was collected through a self-administered electronic questionnaire which was circulated through social media.

Results

The mean ± SD age was reported to be 25.98 ± 12 years. The study found a significant association between blood group B and type 2 diabetes mellitus (p=0.006), whereas a negative association was seen between the blood group O and type 2 diabetes mellitus (p=0.001). It should be noted, however, no significant association was found between the blood groups A and AB and type 2 diabetes mellitus (p>0.05).

Conclusion

The results of this study indicate that there is an association between type 2 diabetes mellitus and the ABO blood group system; a significant association was found between blood group B and risk of type 2 diabetes mellitus (T2DM). Nevertheless, we recommend regular screening for T2DM in individuals with a high-risk profile. Those at risk can adopt measures that are beneficial for them in the long run such as dietary control and physical exercise. Further studies using explorative techniques with a diversified population are recommended.

## Introduction

Diabetes mellitus is a heterogeneous disease characterized by hyperglycemia due to vitiated insulin secretion by the beta cells of the pancreas and/or vitiated insulin action [1,2]. Adult-onset type 2 diabetes mellitus is defined as a chronic hyperglycemic state, characterized by insulin resistance and declining B-cell function, eventually leading to B-cell failure [3]. Even though type 2 diabetes mellitus is a multifactorial disease and is a result of both genetic and environmental factors (i.e., pollution, vitamin D deficiency, and damage to immune cells), regular physical activity can reduce its risk by improving glycemic control [4].

Some common symptoms of type 2 diabetes mellitus include excessive thirst, frequent urination, slow healing of sores, blurred vision, and fatigue. The global incidence of diabetes mellitus has been estimated to be 425 million in 2017, and the prevalence of type 2 diabetes mellitus is around 285 million which is approximated to grow to 438 million by 2030 [2,5]. However, the prevalence of type 2 diabetes mellitus in Pakistan is estimated to be 25 million [6].

Recent literature indicates a correlation between type 2 diabetes mellitus and the ABO blood group system. A study conducted in Turkey reported an increased risk of type 2 diabetes mellitus in participants with type A blood group [7]. A study published in the *Libyan Journal of Medicine* in 2010 was carried out among 70 patients with type 2 diabetes revealed that most participants with blood groups A and O did not have type 2 diabetes mellitus [8]. The A, B, and O blood groups, first identified by Karl Landsteiner, contain two antigens, A and B, and four phenotypes, A, B, O, and AB [9]. ABO antigens are carbohydrate structures bound to glycoproteins and glycolipids [10]. The Rhesus system is found in close association with the ABO blood group system, and Rhesus (or also known as Rh D) is an antigen that is found on the surface of red blood cells. The presence or absence of Rh antigen determines whether a person is Rh+ or Rh- [11,12].

Whether there is a relationship between blood group and the likelihood of acquiring diabetes is still uncertain. Therefore, the current study aimed to evaluate the significance of different blood groups as a predisposing factor for adult-onset diabetes mellitus type 2. 

## Materials and methods

A case-control study was conducted from April 2020 to September 2021 in Karachi, Pakistan. Ethical approval was obtained from the Institutional Review Board (IRB) prior to the study (JSMU/IRB/456). The study employed a nonprobability convenience sampling technique to select the participants. All participants with or without diabetes mellitus type 2 aged 18 or above were included in the study. Individuals who had hemoglobinopathies including thalassemia or sickle cell anemia were excluded from the study. Furthermore, those who were above 60 years and were never checked for diabetes mellitus were also excluded. 

Informed consent was obtained from all the participants. The questionnaires were circulated by the researchers through social media sites such as Facebook, Instagram, WhatsApp, etc. In-person contact with the subject was avoided due to the ongoing pandemic situation. A total of 300 people were approached, of those 200 responded with a response rate of 66.67%. Six questionnaires were incomplete and were excluded from the analysis. One hundred ninety-four patients were included in the study with 97 participants in each group, i.e., case and control. A preformed proforma was used to collect sociodemographic data of the participants. The participant’s blood group, history of diabetes mellitus type 2, and other comorbidities were documented. 

Participants were divided into two groups. Group A had participants with type 2 diabetes mellitus, whereas group B served as the control group. The Statistical Package for the Social Sciences (IBM SPSS, Chicago, USA) was used to analyze the data. All continuous variables were presented as mean and standard deviation, while for all categorical variables, frequency and percentages were evaluated. The Chi-square test was used as a test for significance with significant differences considered at a p-value of 0.05 or less.

## Results

A total of 194 participants with 97 in each group were included in the final analysis. The mean ± SD age was 25.98 ± 12 years. Forty-eight females (24.74%) and 49 males (25.25%) had type 2 diabetes mellitus (T2DM). Family history of T2DM was positive in 120 (61.90%) participants. One hundred twenty-nine participants (66.50%) reported being physically active, and 102 participants (52.60%) consumed a balanced diet. One hundred ten participants (56.70%) had no other comorbidities, and 127 (65.50%) participants never had a blood transfusion (Table 1). There was no statistical difference between case versus control with respect to the demographic profile of participants (p>0.05).

**Table 1:** Demographic Characteristics of Study Participants

Item	n	%
Family history of diabetes		
Yes	120	61.90
No	74	38.10
Gender		
Male	92	47.40
Female	102	52.60
Residence		
Urban	184	94.80
Rural	10	5.20
Employment status		
Government job	10	5.20
Private job	34	17.50
Unemployed	111	57.20
Others	39	20.10
Education status		
Bachelors (graduated)	75	38.70
Intermediate (secondary high school)	106	54.60
Postgraduate or higher	13	6.70
Marital status		
Married	34	17.50
Unmarried	157	80.90
Widowed	3	1.50
Ethnicity		
Urdu speaking	135	69.60
Punjabi	14	7.20
Pashtun	10	5.20
Sindhi	19	9.80
Other	16	8.20
Comorbidity (other than diabetes mellitus)		
Coronary heart disease	6	3.10
Migraine	21	10.80
Myocardial infarction	6	3.10
Renal disease	2	1.00
Hypertension	14	7.20
Ulcer (peptic ulcer)	6	3.10
Others	29	14.90
None	110	56.70
Exercise/physically active		
Yes	129	66.50
No	65	33.50
Ever diagnosed with diabetes mellitus type 2		
Yes	97	50.00
No	97	50.00
Ever donated blood		
Yes	67	34.50
No	127	65.50
Had a blood transfusion		
Yes	16	8.20
No	178	91.80
Blood type		
A	44	22.70
B	88	45.40
AB	23	11.90
O	39	20.10
Daily diet		
Balanced diet	102	52.60
Rich in carbohydrates and fats	84	43.30
Lots of fruits and vegetables	8	4.10

Upon cross-tabulation and application of appropriate Chi-square test, significant associations were found between blood group B (p=0.006) and blood group O (p=0.001) with type 2 diabetes mellitus (T2DM). This implies that patients with blood group B were more frequently diagnosed with T2DM, while individuals with blood group O were less frequently diagnosed with T2DM. It should be noted, however, no significant association was found between the blood groups A (p=0.631) and AB (p=1.000) and T2DM (Table 2).

**Table 2:** Association of Individual Blood Groups With Type 2 Diabetes Mellitus

Blood Group	Patients	Control	P-value
A	21	18	0.631
AB	14	14	1.000
B	57	31	0.006
O	5	34	0.001

## Discussion

In our study, we reported that the majority of the population with blood group B had been diagnosed with type 2 diabetes mellitus (T2DM); however, only a small fraction of people with blood group O had been diagnosed with the disease. Our study coincides with existing literature [13,14]. Zaidi et al. reported a positive association between T2DM and the ABO blood group system; T2DM and the ABO blood group system are interrelated on a genetic immunologic basis; however, the study inferred that the individuals with blood groups type B and A have a higher frequency of T2DM [14]. In 2014, Qureshi and Bhatti found out that the incidence of T2DM among blood B was more common and the incidence of blood group O was the least common [9]. Bener and Yousafzai also supported these findings by concluding that blood group B was more common, whereas blood group O was the least common among diabetic populations [15]. Kamil et al. reported a higher frequency of participants with blood group B who had been diagnosed with T2DM compared with patients who had blood groups A and O [8]. Stern et al. revealed that there were statistically significant correlations between T2DM and RH blood type (p=0.0003) [16].

However, in contrast to the current findings, Alanazi et al. reported inconclusive findings [13]. A study conducted in Rawalpindi, Pakistan, reported that a smaller number of participants with blood groups A and B were seen among diabetic patients and a higher frequency of the blood group AB in the diabetic group [17]. Karagoz et al. explored the relationship between ABO blood groups with gestational diabetes mellitus. The authors revealed that those individuals with blood group AB were at augmented risk of developing gestational diabetes mellitus [18]. Fagherazzi et al. reported that individuals with blood group type O are more prone to developing endocrinological disorders [19]. According to Okon et al., patients with blood group A were more susceptible to T2DM [20]. A study conducted in Muzaffarnagar, India, in 2018 which included a total of 1,316 participants found an increased association of AB and O blood groups with type 2 diabetes mellitus [21]. Abegaz also supported this claim; in their review, they deduced that many studies have found significant relationships between several cancers, metabolic disorders, and other noninfectious disorders [22]. 

Current data on the association between type 2 diabetes mellitus and the ABO blood group system are contradicting as some studies show a positive association, whereas others offer a negative association. It may be noted, however, that the contradictory results can be due to differences in ethnicity and sociodemographic changes affecting the genetic expression of the disease.

The current study serves as a helpful tool for raising awareness among the public regarding the possible association of certain blood group types and T2DM. Those at risk can adopt measures that are beneficial for them in the long run such as dietary control and physical exercise. The current study had some limitations. For instance, the data acquisition was hindered by the ongoing global pandemic and data collection was limited to electronic interviews. Another limitation was the self-reported blood groups and diabetes mellitus diagnosis. Finally, other causes of diabetes mellitus such as the family history of obesity were not taken into consideration; therefore, these factors may play a role in the increased prevalence of T2DM in blood group B participants. Further research exploring how genetic blood composition affects the likelihood of acquiring diabetes mellitus is needed. 

## Conclusions

The results of this study indicate that there is an association between type 2 diabetes mellitus and the ABO blood group system; a significant association was found between blood group B and type 2 diabetes mellitus (T2DM). Nevertheless, we recommend regular screening for T2DM in individuals with a high-risk profile. Those at risk can adopt measures that are beneficial for them in the long run such as dietary control and physical exercise. Further studies using explorative techniques with a diversified population are recommended. 
